# Temporal auditory processing in people exposed to musical instrument practice

**DOI:** 10.1590/2317-1782/20212021256en

**Published:** 2022-08-29

**Authors:** Flavio Van Ryn, Débora Lüders, Raquel Leme Casali, Maria Isabel Ramos do Amaral

**Affiliations:** 1 Departamento de Fonoaudiologia, Universidade Estadual do Centro Oeste – UNICENTRO - Irati (PR), Brasil.; 2 Programa de Pós-graduação Stricto Sensu em Distúrbios da Comunicação, Universidade Tuiuti do Paraná - Curitiba (PR), Brasil.; 3 Departamento de Desenvolvimento Humano e Reabilitação, Faculdade de Ciências Médicas, Universidade Estadual de Campinas – UNICAMP - Campinas (SP), Brasil.

**Keywords:** Auditory Perception, Music, Hearing Tests, Evoked Potentials Auditory, Adult

## Abstract

**Purpose:**

To investigate the influence of musical instrument practice on temporal auditory abilities and on the results of cortical potentials related to auditory events (P300) in a group of young musicians compared to individuals without experience in musical practice.

**Methods:**

This is a prospective cross-sectional observational study. In total, 34 individuals between 18 and 30 years old, of both sexes, took part and were divided in two groups: Group I (GI), composed of musicians (n=16), and Group II (GII), composed of non-musicians (n=18). All participants underwent behavioral evaluation of temporal auditory processing, composed of Duration Pattern Sequence Test (DPS), Pitch Pattern Sequence Test (PPS), Random Gap Detection Test (RGDT) and electrophysiological evaluation – Long Latency Auditory Evoked Potential – P300. GI also answered a specific questionnaire to characterize musical practice.

**Results:**

We observed statistically significant differences with superior performance of GI compared with GII in all behavioral tests (p<0.001*). The groups’ performance was similar regarding the latency and amplitude parameters analyzed from LLAEP-300 data (p>0.05).

**Conclusion:**

The findings show a positive influence of musical practice toward the improvement of auditory abilities of temporal ordering and resolution. All participants presented adequate cortical functioning of the central auditory nervous system, without significant differences between musicians and non-musicians when considering P300 amplitude and latency.

## INTRODUCTION

Greater knowledge about the benefits of musical practice to human development is available nowadays, resulting in an increasing number of people who feel encouraged to study some kind of musical instrument. Recent studies reinforce the evidence that music refines the auditory skills of practitioners given the requirements in musical perception and cognitive demand, contributing to the maturation and development of central auditory nervous system (SNAC) skills throughout life^(1,2)^.

Although the maturational process of SNAC is more evident in the first years of life, it is known that neuronal plasticity does not cease after this phase, and the brain has a great capacity for reorganization that can occur throughout life, influenced by environmental stimuli. Auditory stimulation is thus crucial to strengthen auditory pathways and cortical organization of acoustic representation of sounds, from the first years of life from adulthood to older age^(3)^.

Studies comparing musicians and non-musicians, based on objective methods and neuroimaging exams, demonstrate significant structural differences in cortical motor areas, such as the precentral gyrus, corpus callosum, Heschl’s gyri and in the corticospinal system’s white matter^(4,5)^. Evidence shows that musicians who start their studies earlier and have been inserted longer in musical practice perform better in the synchrony of visual-motor tasks and better integrity of the white matter in the corpus callosum, connecting the premotor and motor cortices^(5)^.

Acoustic interpretation capacity of a signal is known to be improved by musical auditory training. Thus, music is considered a stimulus that favors neuroplasticity and reorganization of cortical maps; music also stimulates cognition and reasoning mechanisms since its practice requires mastery of abstract cognitive processes^(6)^. Therefore, musical stimulation is an adequate practice for the development and stimulation of different abilities, such as sensory, motor and cognitive functions and central auditory processing (CAP) skills^(6,7)^.

Regarding CAP, we highlight the temporal auditory abilities related to the characteristics of duration, organization (ordering in a temporal sequence) and perception of pauses or spectral variations of sound, which are considered the basis for the perception of other auditory processing skills, such as sound localization, figure-ground and integration of acoustic information^(8)^. Behavioral tests standardized by age exist and are applied in clinical practice in the behavioral evaluation of temporal auditory processing (TAP) skills, allowing the measurement of performance in temporal resolution (TR) and temporal ordering (TO) skills^(9,10)^. However, studies on TAP and electrophysiological evaluation of hearing in individuals exposed to musical practice are scarce.

This study aimed to investigate the influence of musical instrument practice on the time auditory abilities and on the results of cortical potentials related to auditory events (P300) in a group of young musicians compared to a group of individuals without experience in musical practice.

## METHODS

### Type of study and study location

This study was developed at the Clinic School of Speech Therapy from State University of Centro-Oeste – UNICENTRO, Paraná, Brazil. This is a prospective, observational, analytical, cross-sectional and quantitative study approved by the Research Ethics Committee of UNICENTRO, under opinion no. 973,331. All participants over 18 years old signed the Informed Consent Form.

### Selection of subjects and characterization of the sample

Individuals aged 18 to 30 years, of both sexes, were selected based on the following inclusion criteria: normal hearing (verified by basic audiological evaluation); normal binaural integration mechanism (verified by the Dichotic Digits Test – DDT) and brainstem integrity (verified using the brainstem auditory evoked response test – ABR).

The exclusion criteria considered individuals from both groups who reported diseases or syndromes that could damage the auditory system, even if progressive and late; with neurological alterations and/or who demonstrated difficulties in understanding the tasks to be performed in the study procedures; were exposed to high sound pressure levels by labor activities.

After applying the inclusion and exclusion criteria, the participants were divided in two groups, matched by gender and age group:

Group I (GI): participants with regular musical practice for at least two years until the date of data collection, without history of chronic otological alterations.

Group II (GII): non-musician participants, without any previous musical practice without complaints and/or history of chronic auditory or otological alterations.

### Prior procedures

Prior to the beginning of data collection, all participants answered an audiological anamnesis and the GI participants also answered an specific questionnaire, with questions regarding the time of musical practice, hours of weekly study and type of instrument. Next, the normality criteria adopted are briefly described regarding the previous procedures applied, following the inclusion criteria described before:

- Basic audiological evaluation: only participants who presented hearing thresholds lower than or equal to 20 dB were included, according to the World Health Organization criteria^(11)^, compatible speech recognition thresholds and speech recognition index above 92%. Regarding acoustic immittance testing, only individuals with peak complicity between -150 to +100 daPa, equivalent volume from 0.3 to 1.6 ml (curve type A) and acoustic reflex from 70 to 100 dB above the hearing threshold for pure tone, at frequencies from 500 to 4000 Hz, were included.
- Dichotic Digit Test (DDT), validated version for Portuguese language^(12)^: applied in the binaural integration stage. Only individuals who presented a percentage of correct answers greater than or equal to 95% in each ear were included.
- Brainstem Auditory Evoked Response (ABR): The equipment used was the Contronic MASBE-ATC plus. The achievement and normality parameters described by Souza et al.^(13)^ were used, with two records on each side to confirm the response, considering the absolute values and interpeak between waves I, III and V.

### Data collection

After selection, the behavioral evaluation of Temporal Auditory Processing was performed in an acoustic booth, with Danplex DA65 audiometer and TDH 39 headphone duly calibrated, consisting of the following procedures:

- Pitch Pattern Sequence Test (PPS) and the Duration Pattern Sequence Test (DPS), Musiek versions^(9)^: applied monophonically at 40 dBNS (sensation level) of intensity based on the average of the tonal threshold of 500, 1000 and 2000 Hz frequencies and in two response modes in each ear: verbal description of the sequence heard (naming) and humming. For the naming stage, normality is considered with at least 76% and 83% of correct answers for the PPS and DPS, respectively^(14)^.
- Random Gap Detection Test (RGDT)^(10)^: binaural presentation, at 50 dBNS, of pure tone pairs at 500, 1000, 2000 and 4000 Hz frequencies, with gap between the two tones that randomly increase or decrease in duration, ranging between 0, 2, 5, 10, 15, 20, 25, 30 and 40 milliseconds (ms) intervals. Individuals were instructed to answer with gestures if they heard one or two tones, that is, whether the gap’s presence was noted. The threshold detection of the gap was calculated individually for each frequency tested, as well as the total test response, using the arithmetic mean of results in the four frequencies evaluated.

Finally, the participants were submitted to the Long Latency Auditory Evoked Potential (LLAEP-P300) study. The P300 was captured in a quiet room and electrically protected using the Contronic MASBE-ATC plus equipment. The participant’s skin was cleaned with alcohol and abrasive paste, conductive gel was then applied and surface electrodes were placed, with active electrodes (Fz) and ground electrodes (Fpz) on the forehead, and reference electrodes in the right (M2) and left (M1) mastoids. The impedance values of the electrodes were verified and must be below 5 kOhms. Monophonic insertion and stimulation headphones were used. The parameters used in the P300 recording included the *tone burst* stimulus, presented monophonically at 75dBNA, presentation speed 1.1 clicks per second, and 300 stimuli in total. The frequent stimulus was presented at 1000 Hz and the rare at 2000 Hz. Of the 300 stimuli presented, 15% to 20% referred to the rare stimulus and the remainder to the frequent stimulus. The high-pass filter was 1 Hz, the low-pass 30 Hz and the analysis window 500 ms, according to the Junqueira and Colafêmia^(15)^ protocol. The variable analyzed was the N1-P2-N2 latency interpeaks and P300 wave. The normality pattern for P300 wave latency was 225 to 365 ms, as proposed by McPherson^(16)^, for the age group of 17 to 30 years.

### Statistical analysis of results

The statistical analysis used the software Statistical Package for the Social Science (SPSS) Version 17. Descriptive statistics, including mean, median and standard deviation to demonstrate the performance in the tests applied in each group, separately and by ear. The test for equality of two proportions verified the homogeneity of the groups in terms of gender and age and the Paired Student’s t test compared the performance of the right and left ears in the auditory tests in each group separately. ANOVA compared the performance of GI to GII in the applied tests. Finally, Pearson’s Correlation was performed to verify the relationship between musical practice and the results of behavioral and electrophysiological evaluation in GI. The significance level adopted in the analyses was 0.05 (5%) and all p-values considered statistically significant were marked with an asterisk (*).

## RESULTS

The sample consisted of 34 subjects, 16 participants (47.05%) from group I (GI), and 18 (52.95%) from group II (GII). GI consisted of 8 (50%) women and 8 (50%) men, the mean age was 21.2 years (±3.1 years) and GII consisted of 8 women (44.40%) and 10 mean (55.60%), of the mean age was 21.5 years (±2.8 years). The groups were considered homogeneous in terms of sex (p=0.746) and age (p=0.803).

The collected data regarding the time and frequency of musical practice were compiled in Table 1. According to the answers obtained, the data were grouped in two categories of answer for each question. The statistics in each category of answer was the same.

**Table 1:** Information on the musical instrument practice of GI members, according to the answers obtained by the questionnaire applied

**Time playing**	**N**	**%**	**P-value**
Up to 10 years old	9	56.30%	0.480
More than 11 years old	7	43.80%	
**Days a week**	**N**	**%**	**P-value**
Up to 4 days	7	43.80%	0.480
More than 5 days	9	56.30%	
**How many hours**	**N**	**%**	**P-value**
Up to 1 hour	10	62.50%	0.157
More than 2 hours	6	37.50%	

Equality Test of Two Proportions

Initially, we compared the performance of the right and left ears in each group separately following the Paired Student’s t test and found statistically significant mean differences between the right and left ears in the PPS in GII (p=0.037*) and DPS in GI (p=0.020) and GII (p=0.017), as well as in wave I of the ABR in both groups (p<0.001)*. Thus, we decided to show the results referring to the comparison of the performance of the groups in the behavioral tests and in the P300 as a function of the right and left ears.

Table 2 shows the comparison of the performance of GI and GII in the PPS and DPS tests and Table 3 refers to the RGDT test. GI presented a performance statistically higher than GII in both temporal ordering tests and in both response modalities (PPS and DPS), as well as in the threshold detection of the gap of all frequencies evaluated by the RGDT and the final mean threshold calculated.

**Table 2:** Mean percentage of correct answers in the Pitch Pattern Sequence Test (PPS) and Duration Pattern Sequence Test (DPS), considering the performance in the right and left ears and the intergroup analysis

**Pitch Pattern Sequence Test**				**N**		**Mean**	**Median**	**SD**		**P-value**
**Naming**		RE	GII	18		87.40%	86.70%	4.60%		<0.001*
			GI	16		97.50%	100.00%	3.90%		
		LE	GII	18		85.40%	83.40%	4.00%		<0.001*
			GI	16		96.50%	96.70%	4.50%		
**Humming**		RE	GII	18		91.50%	90.00%	4.20%		<0.001*
			GI	16		99.20%	100.00%	1.50%		
		LE	GII	18		89.10%	86.70%	4.10%		<0.001*
			GI	16		99.00%	100.00%	2.00%		
**Duration Pattern Sequence Test**					**N**	**Mean**	**Median**		SD	**P-value**
**Naming**	RE		GII		18	85.20%	81.70%		7.80%	<0.001*
			GI		16	100.00%	100.00%		0.00%	
	LE		GII		18	84.80%	80.10%		8.00%	<0.001*
			GI		16	100.00%	100.00%		0.00%	
**Humming**	RE		GII		18	91.10%	90.00%		6.40%	<0.001*
			GI		16	100.00%	100.00%		0.00%	
	LE		GII		18	90.10%	88.40%		6.80%	<0.001*
			GI		16	100.00%	100.00%		0.00%	

Caption: RE = Right Ear; LE = Left Ear; SD = Standad Deviation *Statistically significant value at 5% (p ≤ 0.05). ANOVA test

**Table 3:** Results of the mean threshold in milliseconds, obtained in the application of the RGDT test, by frequency and final result, considering intergroup analysis

**RGDT**		**N**	**Mean**	**Median**	**SD**	**P-value**
**500 Hz**	GII	18	7.06	5	2.80	<0.001*
	GI	16	2.75	2	1.34	
**1 kHz**	GII	18	7.78	10	2.56	<0.001*
	GI	16	3.31	2	1.54	
**2 kHz**	GII	18	8.89	10	2.14	<0.001*
	GI	16	3.81	3.5	2.23	
**4 khz**	GII	18	9.44	10	2.91	<0.001*
	GI	16	4.75	5	1.84	
**Mean**	GII	18	8.29	8.75	1.70	<0.001*
	GI	16	3.66	3.5	1.34	

Caption: Hz = Hertz; SD = Standard Deviation *Statistically significant value at 5% level (p ≤ 0.05). ANOVA test

Table 4 shows the results of the Long Latency Auditory Evoked Potential - P300 from both groups. The statistics in the intergroup comparison was the same.

**Table 4:** Comparison between the mean latencies and interpeaks, in milliseconds, of the P300 wave and N1, P2, N2 complexes in the right and left ears, considering the intergroup analysis

**P 300 (Latency)**				**N**	**Mean**		**Median**			**SD**	**P-value**	
**N1**		RE	GII	18	190.4		187.1			55.9	0.719	
			GI	16	198.3		178.3			71.0		
		LE	GII	18	199.0		206.0			54.8	0.230	
			GI	16	225.3		221.8			70.0		
**P2**		RE	GII	18	218.9		219.3			49.4	0.485	
			GI	16	231.7		216.1			57.0		
		LE	GII	18	210.0		215.5			64.3	0.080	
			GI	16	248.4		236.9			58.6		
**N2**		RE	GII	18	259.2		264.7			36.3	0.451	
			GI	16	270.3		262.2			48.0		
		LE	GII	18	253.1		249.6			38.2	0.062	
			GI	16	280.8		265.3			45.2		
**P 300**		RE	GII	18	316.1		319.6			32.4	0.077	
			GI	16	336.0		330.3			30.8		
		LE	GII	18	313.5		318.9			40.0	0.144	
			GI	16	332.5		324.0			33.1		
**P 300 (Interpeaks)**				**N**		**Mean**		**Median**	**SD**			**P-value**
**N1-P2**	RE		GII	18		28.45		26.49	12.89			0.344
			GI	16		33.43		30.28	17.21			
	LE		GII	18		23.76		17.03	15.15			0.909
			GI	16		23.10		15.14	18.23			
**P2-N2**	RE		GII	18		40.37		48.57	23.89			0.819
			GI	16		38.55		34.69	21.72			
	LE		GII	18		30.27		24.60	13.97			0.727
			GI	16		32.40		22.71	20.97			
**N2 - P300**	RE		GII	18		56.84		58.03	17.67			0.216
			GI	16		65.67		67.49	23.03			
	LE		GII	18		60.48		60.56	19.64			0.212
			GI	16		51.77		54,24	20.16			

Caption: RE = Right Ear; LE = Left Ear; SD = Standad Deviation ANOVA test

Finally, Table 5 demonstrates the correlation analysis between musical instrument practice and the results of DPS, RGDT and electrophysiological behavioral tests. The sample did not present variability of responses in the PPS that allowed correlation analysis, and all participants reached 100% of correct answers in both modalities (naming and humming). For this analysis, we used the grouped data in Table 1 and no significant differences were found.

**Table 5:** Correlation of musical instrument practice with the results of behavioral and electrophysiological auditory tests obtained in GI

				Time playing		Days a week		How many hours	
				Corr (r)	P-value	Corr (r)	P-value	Corr (r)	P-value
	**Duration Pattern Test**	**Naming**	RE	6.00	0.826	-28.50%	0.285	0.121	0.655
			LE	0.60%	0.981	-37.60%	0.152	0.185	0.494
		**Humming**	RE	-25.80%	0.334	-5.00%	0.853	0.257	0.336
			LE	-23.10%	0.389	-18.00%	0.504	0.263	0.325
	**Random Gap Detection Test (RGDT)**	500 Hz		0.00%	1	18.50%	0.494	-0.257	0.336
		1.000 Hz		-16.90%	0.531	-35.50%	0.177	-0.489	0.055
		1.000 Hz		14.30%	0.598	22.50%	0.402	-0.057	0.833
		4.000 Hz		-6.30%	0.818	-1.20%	0.964	-0.116	0.669
		Mean		-1.10%	0.968	23.80%	0.375	-0.269	0.315
**Electrophysiologic Test**	**P 300**	**RE**	N1	22.50%	0.403	10.30%	0.704	-0.355	0.177
			P2	17.40%	0.520	7.60%	0.779	-0.322	0.225
			N2	20.60%	0.445	7.50%	0.784	-0.152	0.573
			P 300	15.40%	0.57	-7.40%	0.786	−0.221	0.412
		**LE**	N1	39.40%	0.131	8.20%	0.764	-0.008	0.977
			P2	37.30%	0.155	-0.60%	0.983	-0.143	0.597
			N2	26.60%	0.320	-18.50%	0.492	-0.300	0.259
			P 300	19.40%	0.472	-11.60%	0.668	-0.241	0.369

Caption: RE = Right Ear; LE = Left Ear; Hz = Hertz; SD = Standard Deviation; Corr (r) = Correlation Coefficient

## DISCUSSION

Musical instrument practice requires refined auditory performance. Musicians need adequate perception of acoustic variations such as volume, duration, timbre and intensity. Thus, we discuss the influence of musical instrument practice on the improvement of temporal auditory abilities and the auditory pathway of the central nervous system (CNS).

Both groups presented mean values, per ear and response modality, within the normality standards for the age group. However, although the variation was within the normal range, GI presented statistically higher performance than GII in the stages of naming and humming in both ears (p<0.001*). These results corroborate other studies with similar methods, in which the performance of musicians was higher and more homogeneous than non-musicians^(17,18)^. The performance within the normal range of both groups indicates that individuals not exposed to musical practice can present some degree of musical perception, but not with the same precision when compared to musicians, since this perception may be directly related to the development and improvement of auditory abilities^(19)^.

Tones are known to be recognized as music or melody, as they are composed of tones of different frequencies and durations in various time orders. The ability to recognize, identify and order acoustic patterns requires several perceptual and cognitive processes that involve the integration of both hemispheres. The left hemisphere is responsible for temporal ordering, linguistic qualification and sequencing of linguistic elements. The right one for the recognition of the acoustic contour and perception of pitch. Memory participates in these auditory processes as a necessary prerequisite for the proper functioning and storage of information during sound processing in the central auditory pathways, allowing the proper reproduction of the sound sequence heard, for example^(8)^. Therefore, exposure to music theory and auditory training are important factors for the good performance to recognize frequency and duration patterns.

As for the better performance of the group of musicians in the temporal resolution ability, this data corroborates other studies in the literature that demonstrate a lower temporal resolution threshold in musicians, evaluated under different acoustic parameters. This difference is attributed to a higher processing speed provided by musical practice^(20,21)^. We highlight that the temporal resolution ability is considered a prerequisite for the processes of reading and writing acquisition, speech perception and good performance as a speaker and listener, since it allows the recognition of speech sounds toward specific characteristics such as changes in duration, pauses and syllable speed^(22)^.

Temporal auditory processing and temporal ordering and resolution skills are thus relevant for speech intelligibility at two levels: suprasegmental (prosodic) and segmental (phonemic). At the segmental level, the speed and rhythm of the syllables influence the lexical and syntactic processing of the language and at a suprasegmental level, duration and gap clues influence the identification of the phoneme^(23)^. Thus, GI’s better performance in the behavioral tests compared to GII reinforces the idea that musical instrument practice can benefit individuals who present complaints or difficulties resulting from alterations in auditory processing.

Exposure to external stimuli is known to influence processes related to neural plasticity, thus favoring a better performance in auditory abilities, especially toward time skills^(6,20,21)^. The results of the present research reinforce the evidence that musical instrument training allows a better perception in the discrimination of temporal pattern sequences and in the ability to solve aspects related to time, since musical practice improves temporal ordering and resolution skills, improving the subject’s auditory acuity.

Although behavioral findings show an improvement in the auditory abilities studied, the electrophysiological evaluation performed according to the p300 cognitive potential demonstrated findings within the normal range in both groups, without statistical differences and mean latency values better in GII when compared to GI (Table 4). P300 latency can be used as a measure of information processing speed in an eccentric paradigm, which can be considered the main parameter for evaluating the response, reflecting the time of evaluation of the rare stimulus presented, the selective care process and/or the updating of working memory. Therefore, some scholars consider this parameter the most reliable indicator since it is difficult to be altered by the subject’s attention^(24)^.

Our findings differ from the initial hypothesis that the best behavioral performance of GI would also be demonstrated in electrophysiological results, as already described in a previous study, in which the authors observed a lower P300 wave latency in the group of musicians toward the group of non-musicians using stimulation without contralateral noise^(25)^. A possible hypothesis for this data may be the fact that in the study cited the authors evaluated a larger sample, which made it possible to evidence behavioral changes from the electrophysiological data. In addition to a smaller sample in our study, it is also necessary to consider the wide range of normality established for the P300 wave. The normality interval established in the literature for P300 latency in the age group evaluated is quite large, ranging from 225 ms to 365 ms, as it considers the maturational process that occurs in the structures of the central auditory pathway as a whole. Therefore, this potential demonstrates a great variability for latency in the comparison between the subjects, in addition to their generating sites, as well as other parameters still being widely discussed in the literature, which may be influenced by numerous other factors present over the maturational course of each individual^(26)^.

Another aspect to be considered for the results is related to the use of the tone burst stimulus. Pure tones are often used clinically to obtain N1-P2-N2 complexes, an exogenous response obtained passively during stimulus presentation; and the P300 wave represents an endogenous response resulting from the cognitive demand required by the oddball paradigm. The tone burst stimulus is composed of acoustically simpler periodic sounds, containing a single frequency component with no variations over time that provides less information about neural function and auditory processing. Other stimuli can be used to obtain the P300 such as the speech stimulus, which has greater acoustic complexity. It is believed that the use of speech stimulus in the sample studied would allow a greater extent of brain regions to be evaluated due to the multiple generating sources in the brain^(16,27,28)^.

Our behavioral data suggests that, when auditory processing skills are evaluated from a functional perspective, the performance of the group of musicians is superior, with more homogeneous results and emphasis the improvement of auditory skills by musical instrument practice. Therefore, we believe that the P300 performed with speech stimulus is better to evaluate the population of musicians.

Finally, we observed that there was no statistically significant correlation between the time of musical instrument practice and the results obtained in the behavioral and electrophysiological tests (p>0.05) (Table 5). This data demonstrates that the time of musical practice did not influence the test results in our sample. However, considering the better performance of GI in temporal auditory processing skills, it can be inferred that, regardless of the time and frequency of musical practice (daily or weekly), auditory processing skills were influenced.

According to the results discussed here, we emphasize the importance of the findings in behavioral evaluation, together with electrophysiological evaluation techniques following auditory evoked potentials. Music is a pleasurable activity and can be used to delineate therapeutic approaches for subjects who present deficit in auditory processing, as they can stimulate the patient in the therapeutic process. Once the benefit of musical practice toward auditory processing is verified, especially the time aspects, regardless of the weekly frequency and time of study, this practice should be valued and implemented as a habit in peoples’ lives, either in schools or at home.

## CONCLUSION

Based on the findings, we conclude that the musicians presented statistically higher results to non-musicians in the behavioral tests of temporal resolution and ordering, demonstrating a positive influence of musical practice toward the improvement of temporal auditory abilities, regardless of the time and frequency of musical practice reported. Both groups presented normal performance in the Long Latency Auditory Evoked Potential - P300, and no significant intergroup differences were found.
