# Supplementary material for: Temporal auditory processing in people exposed to musical instrument practice
Source: Codas. 2022 Aug 29;34(6):e20210256. doi: 10.1590/2317-1782/20212021256en (PMC9886294; doi:10.1590/2317-1782/20212021256en)
Supplement: Translation

# Translation: Processamento auditivo temporal em indivíduos expostos à prática musical instrumental

**DOI:** 10.1590/2317-1782/20212021256pt

**Authors:** Flavio Van Ryn, Débora Lüders, Raquel Leme Casali, Maria Isabel Ramos do Amaral

**Affiliations:** 1 Departamento de Fonoaudiologia, Universidade Estadual do Centro Oeste – UNICENTRO - Irati (PR), Brasil.; 2 Programa de Pós-graduação Stricto Sensu em Distúrbios da Comunicação, Universidade Tuiuti do Paraná - Curitiba (PR), Brasil.; 3 Departamento de Desenvolvimento Humano e Reabilitação, Faculdade de Ciências Médicas, Universidade Estadual de Campinas – UNICAMP - Campinas (SP), Brasil.

**Keywords:** Percepção Auditiva, Música, Testes Auditivos, Potenciais Evocados Auditivos, Adulto

## Abstract

**Objetivo:**

Investigar a influência da prática musical instrumental nas habilidades auditivas temporais e nos resultados de potenciais corticais relacionados a eventos auditivos (P300) em um grupo de jovens músicos em comparação com indivíduos sem experiência prática musical.

**Método:**

Trata-se de um estudo prospectivo, observacional, analítico e transversal. Participaram 34 indivíduos entre 18 a 30 anos, de ambos os sexos, divididos em dois grupos: Grupo I (GI), composto por indivíduos músicos (n=16) e Grupo II (GII), composto por indivíduos não músicos (n=18). Todos os participantes realizaram avaliação comportamental do processamento auditivo temporal, composta pelos testes de Padrão de Duração (TPD), Padrão de Frequência (TPF), *Random Gap Detection* (RGDT) e avaliação eletrofisiológica - Potencial Evocado Auditivo de Longa Latência (PEALL) - P300. O GI respondeu também a um questionário específico para caracterização da prática musical.

**Resultados:**

Foram observadas diferenças estatisticamente significantes com desempenho superior do GI em relação ao GII em todos os testes comportamentais aplicados (p<0,001*). Não foram encontradas diferenças significantes entre os grupos com relação aos parâmetros de latência e amplitude analisados a partir da obtenção do PEALL-300 (p>0,05).

**Conclusão:**

Os achados demonstraram influência positiva da prática musical em relação ao aprimoramento de habilidades auditivas de ordenação e resolução temporal. Todos os participantes apresentaram adequado funcionamento cortical do sistema nervoso auditivo central, sem diferenças significantes entre músicos e não músicos nos parâmetros de amplitude e latência do P300.

## INTRODUÇÃO

Atualmente há um maior conhecimento a respeito dos benefícios que a prática musical traz para o desenvolvimento humano, conhecimento este que vem refletindo em um número cada vez maior de pessoas que são incentivadas a estudar algum tipo de instrumento musical. Estudos recentes reforçam as evidencias de que a música propicia ao indivíduo habilidades auditivas mais refinadas em função do que exige em termos de percepção musical e demanda cognitiva, contribuindo, portanto, no processo de maturação e desenvolvimento de habilidades do sistema nervoso auditivo central (SNAC) ao longo de toda a vida^(1,2)^.

Apesar do processo maturacional do SNAC ser mais evidente nos primeiros anos de vida, sabe-se que a plasticidade neuronal não cessa após essa fase, e o cérebro possui uma grande capacidade de reorganização que pode ocorrer ao longo de toda a vida, influenciada pelos estímulos do meio ambiente. Portanto, entende-se que a estimulação auditiva é crucial para o fortalecimento das vias auditivas e organização cortical da representação acústica dos sons, desde os primeiros anos de vida até a idade adulta e até mesmo idosa^(3)^.

Estudos comparando músicos e não músicos, embasadas em métodos objetivos e exames de neuroimagem, demonstram diferenças estruturais significativas em áreas motoras corticais, como o giro pré-central, corpo caloso, o giro de *Heschl* e na substância branca do sistema córtico-espinal^(4,5)^. As evidencias mostram que músicos que iniciam seus estudos mais cedo e estão inseridos há mais tempo na prática musical apresentam melhor desempenho na sincronia de tarefas viso-motoras e melhor integridade da substância branca no corpo caloso, conectando os córtex pré-motor e motor^(5)^.

Sabe-se que a capacidade de interpretação acústica do sinal pode ser aprimorada pelo treinamento auditivo musical. Desta forma, a música é considerada um estímulo que favorece a neuroplasticidade e reorganização de mapas corticais, estimulando também a cognição, uma vez que a prática musical requer o domínio de processos cognitivos abstratos e estimula os mecanismos de raciocínio^(6)^. Sendo assim, a estimulação musical é apontada como uma prática adequada para o desenvolvimento e estimulação de diferentes habilidades, tais como funções sensoriais, motoras, cognitivas e as habilidades do processamento auditivo central (PAC)^(6,7)^.

Especificamente em relação ao PAC, destacam-se as habilidades auditivas temporais, relacionadas com as características de duração, organização (ordenação em uma sequência temporal) e percepção de pausas ou variações espectrais do som, as quais são consideradas a base para a percepção das demais habilidades do processamento auditivo, tais como a localização sonora, figura-fundo e integração de informações acústicas^(8)^. Os testes comportamentais disponíveis e padronizados por faixa etária para serem aplicados na prática clínica na avaliação comportamental das habilidades do processamento auditivo temporal (PAT) possibilitam a mensuração do desempenho nas habilidades de resolução temporal (RT) e ordenação temporal (OT)^(9,10)^ e há uma escassez de estudos envolvendo o processamento auditivo temporal e avaliação eletrofisiológica da audição em indivíduos expostos a prática musical.

Diante disso, essa pesquisa teve como objetivo investigar a influência da prática musical instrumental nas habilidades auditivas temporais e nos resultados de potenciais corticais relacionados a eventos auditivos (P300) em um grupo de jovens músicos em comparação com um grupo de indivíduos sem experiência prática musical.

## MÉTODO

### Tipo de estudo e local do estudo

O presente trabalho foi desenvolvido na Clínica Escola de Fonoaudiologia da Universidade Estadual do Centro-Oeste – UNICENTRO, Paraná. Trata-se de um estudo prospectivo, observacional, analítico, transversal e caráter quantitativo, aprovado pelo Comitê de Ética em Pesquisa da UNICENTRO, sob o parecer nº 973.331. Todos os participantes maiores de 18 anos assinaram o Termo de Consentimento Livre e Esclarecido.

### Seleção dos sujeitos e caracterização da amostra

Foram selecionados indivíduos na faixa etária de 18 a 30 anos, de ambos os sexos, com base nos seguintes critérios de inclusão: audição normal (verificada por avaliação audiológica básica); mecanismo de integração binaural normal (verificada pelo teste Dicótico de Dígitos -TDD) e integridade do tronco encefálico (verificada a partir do Potencial Evocado Auditivo de Tronco Encefálico - PEATE).

Foram excluídos os indivíduos, de ambos os grupos, que apresentassem relato de doenças ou síndromes que podem cursar com possíveis danos ao sistema auditivo, mesmo que progressivos e tardios; com alterações neurológicas e/ou que demonstrassem dificuldades de compreensão das tarefas a serem executadas nos procedimentos do estudo; relato de exposição a níveis de pressão sonora elevados por outras atividades ocupacionais.

Aplicados os critérios de inclusão e exclusão, os participantes foram divididos em dois grupos, pareados por sexo e faixa etária:

Grupo I (GI): participantes com prática musical regular há pelo menos dois anos até a data da coleta de dados, sem histórico de alterações otológicas crônicas.

Grupo II (GII): participantes não músicos, sem qualquer prática musical prévia sem queixas e/ou histórico de alterações auditivas ou otológicas crônicas.

### Procedimentos prévios

Previamente ao início da coleta de dados, todos os participantes responderam a uma anamnese audiológica e os participantes do GI responderam ainda a um questionário especifico, com perguntas referentes ao tempo de prática musical, horas de estudo semanal e tipo de instrumento percutido. A seguir, serão brevemente descritos os critérios de normalidade adotados referente aos procedimentos prévios aplicados, seguindo os critérios de inclusão descritos anteriormente:

-Avaliação audiológica básica: foram incluídos apenas participantes que apresentaram limiares auditivos menores ou iguais a 20dB, segundo critério da Organização Mundial da Saúde^(11)^, limiares de reconhecimento de fala compatíveis e índice de reconhecimento de fala acima de 92%. Com relação as medidas de imitância acústica, foram incluídos apenas os indivíduos com pico de máxima compliância entre -150 a +100daPa, volume equivalente de 0,3 a 1,6 ml (curva tipo A) e reflexo acústico de 70 a 100dB acima do limiar de audibilidade para tom puro, nas frequências de 500 a 4000Hz.
-Teste Dicótico de Dígitos (TDD), versão validada para o Português^(12)^: aplicado na etapa de integração binaural. Foram incluídos apenas os indivíduos que apresentaram porcentagem de acertos maior ou igual a 95% em cada orelha.

-Potencial Evocado Auditivo de Tronco Encefálico (PEATE) – O equipamento utilizado foi o *Contronic MASBE-ATC plus*. Foram considerados os parâmetros de realização e de normalidade descritos por Souza et al.^(13)^, a partir de dois registros de cada lado para confirmar a existência de resposta, considerando os valores absolutos e interpicos entre as ondas I, III e V.

### Coleta de dados

Após seleção, a avaliação comportamental do Processamento Auditivo Temporal foi realizada em cabina acústica, com audiômetro Danplex DA65 e fone TDAH 39 devidamente calibrados, sendo composta pelos seguintes procedimentos:

- Teste de Padrão de Frequência (TPF – *Pitch Pattern Sequence Test) e* Teste de Padrão de Duração (TPD *- Duration Pattern Sequence Test) –* versões Musiek^(9)^: aplicados de forma monoaural a uma intensidade de 40dBNS (nível de sensação) a partir da média dos limiares tonais das frequências de 500, 1000 e 2000 Hz e em duas modalidades de resposta em cada orelha: descrição verbal da sequência ouvida (nomeação) e murmúrio (*humming)*. Como critério de normalidade, com base na etapa de nomeação, considera-se mínimo de 76% de acertos para o TPF e mínimo de 83% de acertos para o TPD^(14)^.
- Teste de Detecção de Intervalo no Silêncio (RGDT – *Randon Gap Detection Test*)^(10)^: apresentação binaural, a 50 dBNS, de pares de tons puros nas frequências de 500, 1000, 2000 e 4000 Hz, com intervalos (*Gap*) entre os dois tons que aumentam ou diminuem de duração aleatoriamente, variando entre intervalos de 0, 2, 5, 10, 15, 20, 25, 30 e 40 milissegundos (ms). O indivíduo foi orientado a responder gestualmente se ouviu um ou dois tons, ou seja, se notou ou não a presença do *gap.* Calculou-se o limiar de detecção de gap individualmente para cada frequência testada, bem como a resposta total do teste, por meio da média aritmética de resultados nas quatro frequências avaliadas.

Por fim, os participantes foram submetidos a pesquisa do Potencial Evocado Auditivo de Longa Latência (PEALL-P300). A captação do P300 foi realizada em sala silenciosa e protegida eletricamente utilizando o equipamento Contronic *MASBE-ATC plus*. Após limpeza da pele com álcool e pasta abrasiva e aplicação do gel condutor, foram colocados eletrodos de superfície, sendo os eletrodos ativo (Fz) e o terra (Fpz) na fronte, e os eletrodos de referência nas mastóides direita (M2) e esquerda (M1). Os valores de impedância dos eletrodos foram verificados, devendo situar-se abaixo de 5 kOhms. Foram utilizados fones de inserção e estimulação monoaural. Os parâmetros utilizados no registro do P300 incluíram o estímulo “*tone burst”,* apresentado monoauralmente a 75dBNA, em velocidade de apresentação de 1,1 cliques por segundo, sendo empregado um total de 300 estímulos. O estímulo frequente foi apresentado a 1000 Hz e o raro a 2000 Hz. Dos 300 estímulos apresentados, 15% a 20% referiam-se ao estímulo raro e o restante ao estímulo frequente. O filtro passa-alto foi de 1 Hz, o passa-baixo de 30 Hz e a janela de análise de 500 ms, segundo o protocolo de Junqueira e Colafêmina^(15)^. A variável analisada foi a latência dos interpicos N1-P2-N2 e onda P300. O padrão de normalidade para a latência da onda P300 foi de 225 a 365ms, conforme proposto por McPherson^(16)^, para faixa etária de 17 a 30 anos.

### Análise estatística dos resultados

A análise estatística foi realizada por meio do *software “Statistical Package for the Social Science”* (SPSS) Versão 17. A estatística descritiva, incluindo média, mediana e desvio padrão foi realizada para demonstrar o desempenho nos testes aplicados em cada grupo, separadamente e por orelha. O Teste de Igualdade de duas proporções verificou a homogeneidade dos grupos quanto ao sexo e idade e o teste *T Student Pareado* comparou o desempenho das orelhas direita e esquerda nos testes auditivos, em cada grupo separadamente. A ANOVA comparou o desempenho do GI em relação ao GII nos testes aplicados. E por fim, a Correlação de Pearson foi realizada para verificar a relação entre a prática musical e os resultados da avaliação comportamental e eletrofisiológica, no GI. O nível de significância adotado nas análises foi de 0,05 (5%) e todos os p-valores considerados estatisticamente significantes foram assinalados com asterisco (*).

## RESULTADOS

A amostra foi constituída por 34 sujeitos, sendo 16 participantes (47,05%) do grupo I (GI), e 18 (52,95%) do grupo II (GII). O GI foi constituído por 8 (50%) sujeitos do sexo feminino e 8 (50%) do sexo masculino com idade média de 21,2 anos (+ 3,1anos) e o GI foi constituído por 8 sujeitos do sexo feminino (44,40%) e 10 sujeitos do sexo masculino (55,60%) com idade média de 21,5 anos (+2,8anos). Os grupos foram considerados homogêneos quanto ao sexo (p=0,746) e idade (p=0,803).

Os dados coletados referentes ao tempo e frequência da prática musical foram compilados na Tabela 1. Segundo as respostas obtidas, os dados foram agrupados em duas categorias de resposta para cada pergunta. Não houve diferença estatística entre as categorias de respostas.

**Tabela 1:** Informações sobre a prática musical instrumental dos sujeitos do GI, segundo as respostas obtidas pelo questionário aplicado

Tempo que toca	N	%	P-valor
Até 10 anos	9	56,30%	0,480
Mais de 11 anos	7	43,80%	
**Dias por semana**	**N**	**%**	**P-valor**
Até 4 dias	7	43,80%	0,480
Mais de 5 dias	9	56,30%	
**Quantas horas**	**N**	**%**	**P-valor**
Até 1 hora	10	62,50%	0,157
Mais de 2 horas	6	37,50%	

Teste de Igualdade de Duas Proporções

Inicialmente, o desempenho das orelhas direita e esquerda foi comparado em cada grupo separadamente por meio do teste *T Student Pareado* e foram encontradas diferenças médias estatisticamente significantes entre a orelha direita e esquerda no TPF no GII (p=0,037*) e TPD no GI (p=0,020) e GII (p=0,017), assim como na onda I do PEATE em ambos os grupos (p<0,001)*. Desta forma, optou-se por apresentar os resultados referentes à comparação do desempenho dos grupos nos testes comportamentais e também no P300 em função das orelhas direita e esquerda.

A Tabela 2 demonstra a comparação do desempenho dos grupos GI e GII nos testes TPF e TPD e a Tabela 3 refere-se ao teste RGDT. O GI apresentou desempenho estatisticamente superior ao GII em ambos os testes de ordenação temporal e nas duas modalidades de resposta (TPF e TPD), assim como no limiar de detecção de *gap* de todas as frequências avaliadas pelo RGDT e limiar médio final calculado.

**Tabela 2:** Porcentagem média de acertos no Teste de Padrão de Duração (TPD) e Teste Padrão de Frequência (TPF), considerando o desempenho nas orelhas direita e esquerda e a análise intergrupo

Teste de Padrão de Duração				N		Média	Mediana	DP		P-valor
Nomeação		OD	GII	18		87,40%	86,70%	4,60%		<0,001*
			GI	16		97,50%	100,00%	3,90%		
		OE	GII	18		85,40%	83,40%	4,00%		<0,001*
			GI	16		96,50%	96,70%	4,50%		
*Humming*		OD	GII	18		91,50%	90,00%	4,20%		<0,001*
			GI	16		99,20%	100,00%	1,50%		
		OE	GII	18		89,10%	86,70%	4,10%		<0,001*
			GI	16		99,00%	100,00%	2,00%		
Teste de Padrão de Frequência					N	Média	Mediana		DP	P-valor
Nomeação	OD		GII		18	85,20%	81,70%		7,80%	<0,001*
			GI		16	100,00%	100,00%		0,00%	
	OE		GII		18	84,80%	80,10%		8,00%	<0,001*
			GI		16	100,00%	100,00%		0,00%	
*Humming*	OD		GII		18	91,10%	90,00%		6,40%	<0,001*
			GI		16	100,00%	100,00%		0,00%	
	OE		GII		18	90,10%	88,40%		6,80%	<0,001*
			GI		16	100,00%	100,00%		0,00%	

Legenda: OD = Orelha Direita; OE = Orelha Esquerda; DP = Desvio Padrão *Valor estatisticamente significante no nível de 5% (p ≤ 0,05). Teste ANOVA

**Tabela 3:** Resultados do limiar médio em milissegundos, obtido na aplicação do teste RGDT, por frequência e resultado final, considerando análise intergrupo

RGDT		N	Média	Mediana	DP	P-valor
500 Hz	GII	18	7,06	5	2,80	<0,001*
	GI	16	2,75	2	1,34	
1kHz	GII	18	7,78	10	2,56	<0,001*
	GI	16	3,31	2	1,54	
2kHz	GII	18	8,89	10	2,14	<0,001*
	GI	16	3,81	3,5	2,23	
4khz	GII	18	9,44	10	2,91	<0,001*
	GI	16	4,75	5	1,84	
Média	GII	18	8,29	8,75	1,70	<0,001*
	GI	16	3,66	3,5	1,34	

Legenda: Hz = Hertz; DP = Desvio Padrão *Valor estatisticamente significante no nível de 5% (p ≤ 0,05). Teste ANOVA

A Tabela 4 demonstra os resultados do Potencial Evocado Auditivo de Longa Latência – P300, de ambos os grupos, sendo que não foram encontradas diferenças estatisticamente significantes na comparação intergrupos.

**Tabela 4:** Comparação entre a média das latências e interpicos, em milissegundos, da onda P300 e complexos N1, P2, N2 nas orelhas direita e esquerda, considerando a análise intergrupo

P 300 (Latência)				N	Média		Mediana			DP	P-valor	
N1		OD	GII	18	190,4		187,1			55,9	0,719	
			GI	16	198,3		178,3			71,0		
		OE	GII	18	199,0		206,0			54,8	0,230	
			GI	16	225,3		221,8			70,0		
P2		OD	GII	18	218,9		219,3			49,4	0,485	
			GI	16	231,7		216,1			57,0		
		OE	GII	18	210,0		215,5			64,3	0,080	
			GI	16	248,4		236,9			58,6		
N2		OD	GII	18	259,2		264,7			36,3	0,451	
			GI	16	270,3		262,2			48,0		
		OE	GII	18	253,1		249,6			38,2	0,062	
			GI	16	280,8		265,3			45,2		
P 300		OD	GII	18	316,1		319,6			32,4	0,077	
			GI	16	336,0		330,3			30,8		
		OE	GII	18	313,5		318,9			40,0	0,144	
			GI	16	332,5		324,0			33,1		
P 300 (Interpicos)				N		Média		Mediana	DP			P-valor
N1-P2	OD		GII	18		28,45		26,49	12,89			0,344
			GI	16		33,43		30,28	17,21			
	OE		GII	18		23,76		17,03	15,15			0,909
			GI	16		23,10		15,14	18,23			
P2-N2	OD		GII	18		40,37		48,57	23,89			0,819
			GI	16		38,55		34,69	21,72			
	OE		GII	18		30,27		24,60	13,97			0,727
			GI	16		32,40		22,71	20,97			
N2 - P300	OD		GII	18		56,84		58,03	17,67			0,216
			GI	16		65,67		67,49	23,03			
	OE		GII	18		60,48		60,56	19,64			0,212
			GI	16		51,77		54,24	20,16			

Legenda: OD = Orelha Direita; OE = Orelha Esquerda; DP = Desvio Padrão Teste ANOVA

Por fim, a Tabela 5 demonstra a análise de correlação entre a prática musical instrumental e os resultados dos testes comportamentais TPD e RGDT e eletrofisiológicos. No TPF, a amostra não apresentou variabilidade de respostas que permitissem a análise de correlação, sendo que todos os participantes atingiram 100% de acertos em ambas as modalidades (nomeação e *humming*). Para esta análise, foram utilizados os dados agrupados demonstrado na Tabela 1 e não foram encontradas diferenças significantes.

**Tabela 5:** Correlação da prática musical instrumental com os resultados dos testes auditivos comportamentais e eletrofisiológicos obtidos no GI

				**Tempo que toca**		**Dias por semana**		**Quantas horas**	
				Corr (r)	P-valor	Corr (r)	P-valor	Corr (r)	P-valor
	**Teste de Padrão de Duração**	**Nomeação**	OD	6,00%	0,826	-28,50%	0,285	0,121	0,655
			OE	0,60%	0,981	-37,60%	0,152	0,185	0,494
		**Humming**	OD	-25,80%	0,334	-5,00%	0,853	0,257	0,336
			OE	-23,10%	0,389	-18,00%	0,504	0,263	0,325
	**Randon Gap Detection Test (RGDT)**	500 Hz		0,00%	1	18,50%	0,494	-0,257	0,336
		1.000 Hz		-16,90%	0,531	35,50%	0,177	-0,489	0,055
		2.000 Hz		14,30%	0,598	22,50%	0,402	-0,057	0,833
		4.000 Hz		-6,30%	0,818	-1,20%	0,964	-0,116	0,669
		Média		-1,10%	0,968	23,80%	0,375	-0,269	0,315
**Teste Eletrofisiologico**	**P 300**	**OD**	N1	22,50%	0,403	10,30%	0,704	-0,355	0,177
			P2	17,40%	0,520	7,60%	0,779	-0,322	0,225
			N2	20,60%	0,445	7,50%	0,784	-0,152	0,573
			P 300	15,40%	0,57	-7,40%	0,786	-0,221	0,412
		**OE**	N1	39,40%	0,131	8,20%	0,764	-0,008	0,977
			P2	37,30%	0,155	-0,60%	0,983	-0,143	0,597
			N2	26,60%	0,320	-18,50%	0,492	-0,300	0,259
			P 300	19,40%	0,472	-11,60%	0,668	-0,241	0,369

Legenda: OD = Orelha Direita; OE = Orelha Esquerda; Hz = Hertz; DP = Desvio Padrão; Corr (r) = Coeficiente de Correlação

## DISCUSSÃO

A prática musical instrumental requer um desempenho auditivo refinado. O músico precisa ter uma adequada percepção das variações acústicas como altura, duração, timbre e intensidade. Sendo assim, o presente trabalho discute a influência da prática musical instrumental no aprimoramento das habilidades auditivas temporais e sob a via auditiva do sistema nervoso central (SNC).

Nos testes comportamentais de ordenação temporal, ambos os grupos apresentaram valores médios, por orelha e por modalidade de resposta, dentro dos padrões de normalidade para a faixa etária. Porém observa-se que mesmo esta variação estando dentro da normalidade, o GI apresentou desempenho estatisticamente superior em relação ao GII nas etapas de nomeação e *humming* nas duas orelhas (p<0,001*). Tais resultados corroboram com outros trabalhos com métodos semelhantes em que o desempenho do grupo de músicos foi superior e mais homogêneo em relação ao grupo de não-músicos^(17,18)^. O desempenho dentro da normalidade de ambos os grupos indica que indivíduos não expostos a pratica musical podem ser capazes de apresentar algum grau de percepção musical, porém não com a mesma precisão quando comparado a músicos, uma vez que essa percepção pode estar diretamente relacionada com o desenvolvimento e aprimoramento de habilidades auditivas^(19)^.

Sabe-se que os padrões tonais são reconhecidos como música ou melodia, pois são compostos por tons de diferentes frequências e durações em ordens temporais diversas. A habilidade de reconhecer, identificar e ordenar padrões acústicos requer vários processos perceptuais e cognitivos que envolvem a integração de ambos os hemisférios. O hemisfério esquerdo é responsável pela ordenação temporal, qualificação linguística, sequenciação dos elementos linguísticos e o hemisfério direito pelo reconhecimento do contorno acústico e percepção do *Pitch.* Pode-se mencionar ainda a participação da memória nestes processos auditivos, como um pré requisito necessário para o adequado funcionamento e armazenamento das informações ao longo do processamento do som nas vias auditivas centrais, permitindo, por exemplo, a reprodução adequada da sequência sonora ouvida^(8)^. Portanto, a exposição à teoria musical e ao treinamento auditivo são fatores importantes para o bom desempenho nessa tarefa de reconhecer padrões de frequência e duração.

Quanto ao melhor desempenho do grupo de músicos na habilidade de resolução temporal, tal dado corrobora com outros estudos na literatura que demonstram menor limiar de resolução temporal em músicos, avaliado sob diferentes parâmetros acústicos, sendo esta diferença atribuída a uma maior velocidade de processamento proporcionada pela prática musical^(20,21)^. É importante destacar que a habilidade de resolução temporal é considerada pré-requisito, tanto para os processos de aquisição da leitura e escrita, como para a percepção de fala e o bom desempenho enquanto falante e ouvinte, uma vez que possibilita o reconhecimento dos sons da fala em relação a características específicas, tais como mudanças na duração, pausas e velocidade da sílaba^(22)^.

Desta forma, as habilidades do processamento auditivo temporal, ordenação e resolução temporal, são relevantes para a inteligibilidade da fala em dois níveis: em nível suprassegmental (prosódico) e segmental (fonêmico). No nível segmental, a velocidade e o ritmo das sílabas influenciam o processamento léxico e sintático da linguagem e em um nível suprassegmental, pistas de duração e de *gap* influenciam a identificação do fonema^(23)^. Sendo assim, a melhor performance nos testes comportamentais do GI em relação ao GII reforça a ideia de que a prática musical instrumental pode ser benéfica ao indivíduo que apresente queixas ou dificuldades decorrentes de alterações no processamento auditivo.

Sabe-se que a exposição aos estímulos externos exerce influência sobre processos relacionados à plasticidade neural, favorecendo, assim, um melhor desempenho nas habilidades auditivas, especialmente com relação às habilidades temporais^(6,20,21)^. Os resultados decorrentes da presente pesquisa reforçam as evidências de que o treinamento musical instrumental possibilita uma melhor percepção na discriminação de sequências de padrões temporais e na capacidade de resolver aspectos relacionados ao tempo, visto que a prática musical aprimora as habilidades de ordenação e resolução temporal, possibilitando ao sujeito uma acuidade auditiva mais acurada.

Apesar de os achados comportamentais evidenciarem um aprimoramento nas habilidades auditivas estudadas, a avaliação eletrofisiológica realizada por meio do potencial cognitivo P300 demonstrou achados dentro da normalidade em ambos os grupos, sem diferenças estatísticas e valores médios da latência melhores no GII quando comparado ao GI (Tabela 4). A latência do P300 pode ser utilizada como uma medida da velocidade de processamento da informação em um paradigma excêntrico, podendo ser considerada o principal parâmetro de avaliação da resposta, reflete o tempo de avaliação do estímulo raro apresentado, o processo de atenção seletiva e/ou a atualização da memória de trabalho. Portanto, esse parâmetro é considerado por alguns estudiosos como sendo o indicador mais confiável, visto que esta é difícil de ser alterada em função da atenção do sujeito^(24)^.

Os nossos achados diferem da hipótese inicial de que o melhor desempenho comportamental do GI também seria demonstrado em resultados eletrofisiológicos, assim como já descrito em estudo prévio, no qual os autores observaram média de latência da onda P300 menor no grupo de músicos em relação ao grupo de não músicos utilizando a estimulação sem ruído contralateral^(25)^. Uma possível hipótese para esse dado pode ser o fato de que no estudo citado os autores avaliaram uma amostra maior, o que possibilitou evidenciar as mudanças comportamentais a partir do dado eletrofisiológico. Além de uma amostra mais reduzida em nosso estudo, é necessário também considerar a ampla faixa de normalidade estabelecida para a onda P300. O intervalo de normalidade estabelecido na literatura para a latência do P300 na faixa etária avaliada é bastante extenso, podendo variar de 225ms a 365ms, pois considera o processo maturacional que ocorre nas estruturas da via auditiva central como um todo. Portanto, esse potencial demonstra uma grande variabilidade para a latência na comparação entre os sujeitos, além de seus sítios geradores, bem como outros parâmetros ainda serem amplamente discutidos na literatura, os quais podem sofrer influência de inúmeros outros fatores presentes ao longo do curso maturacional de cada indivíduo^(26)^.

Um outro aspecto a ser considerado para os resultados encontrados, relaciona-se com o uso do estímulo “*tone burst*”. Os tons puros são mais frequentemente utilizados clinicamente para a obtenção dos complexos N1-P2-N2, resposta exógena obtida de forma passiva durante a apresentação do estímulo; e a onda P300 representa uma resposta endógena resultante da demanda cognitiva exigida pelo paradigma *oddball*. O estímulo *tone burst* é composto por sons periódicos acusticamente mais simples, contendo um único componente de frequência, não havendo variações ao longo do tempo, o que fornece menos informações sobre a função neural e processamento auditivo. Outros estímulos podem ser utilizados para obtenção do P300, como o estímulo de fala, o qual possui uma maior complexidade acústica. Acredita-se que o uso do estimulo de fala na amostra estudada permitiria com que uma maior extensão das regiões cerebrais fosse avaliada devido às múltiplas fontes geradoras no cérebro^(16,27,28)^.

Em nossos achados, o dado comportamental encontrado sugere que, quando as habilidades do processamento auditivo são avaliadas sob uma perspectiva funcional, o desempenho do grupo de músicos é superior, apresentando resultados mais homogêneos e ressaltando o aprimoramento das habilidades auditivas pela prática musical instrumental. Portanto, acreditamos que o P300 realizado com estímulo de fala seja melhor para avaliar a população de músicos.

Por fim, foi possível observar que não houve correlação estatisticamente significante entre o tempo de prática musical instrumental e os resultados obtidos nos testes comportamentais e eletrofisiológicos (p>0,05) (Tabela 5). Este dado demonstra que o tempo de prática musical não influenciou nos resultados dos testes em nossa amostra, porém, levando em conta o melhor desempenho do GI nas habilidades do processamento auditivo temporal, pode-se inferir que, independentemente do tempo e da frequência de prática musical (diária ou semanal), esta influenciou no aprimoramento das habilidades do processamento auditivo.

Diante dos resultados aqui discutidos, ressalta-se a importância dos achados da avaliação comportamental, aliados às técnicas de avaliação eletrofisiológica por meio dos potenciais evocados auditivos. A música é uma atividade prazerosa, podendo ser utilizada para delinear abordagens terapêuticas para sujeitos que apresentem déficit no processamento auditivo, pois podem estimular o paciente no processo terapêutico. Uma vez constatado o benefício da prática musical com relação ao processamento auditivo, especialmente aos aspectos temporais, independente da frequência semanal e tempo de estudo, tal prática deve ser valorizada e implementada como um hábito na vida do sujeito, seja em escolas ou em casa.

## CONCLUSÃO

Com base nos achados, é possível concluir que os músicos apresentaram resultados estatisticamente superiores aos não músicos nos testes comportamentais de resolução e ordenação temporal, demonstrando influência positiva da prática musical em relação ao aprimoramento de habilidades auditivas temporais, independentemente do tempo e frequência de prática musical relatados. Ambos os grupos apresentaram desempenho dentro da normalidade no potencial auditivo evocado de longa latência- P300, não tendo sido evidenciadas diferenças significantes intergrupos.
